# Genome Survey of Male *Rana dybowskii* to Further Understand the Sex Determination Mechanism

**DOI:** 10.3390/ani14202968

**Published:** 2024-10-14

**Authors:** Yuan Xu, Hanyu Liu, Xinshuai Jiang, Xinning Zhang, Jiayu Liu, Yaguang Tian, Xiujuan Bai, Shiquan Cui, Shengwei Di

**Affiliations:** College of Animal Science and Technology, Northeast Agricultural University, No. 600 Changjiang Road, Xiangfang District, Harbin 150030, China; yuanxu@neau.edu.cn (Y.X.); s230501039@neau.edu.cn (H.L.); s230501037@neau.edu.cn (X.J.); zxn0502104@163.com (X.Z.); y1719511587@163.com (J.L.); tianyaguang2011@163.com (Y.T.); bxiujuan630306@163.com (X.B.)

**Keywords:** *Rana dybowskii*, genome survey, next-generation sequencing technology, sex determination

## Abstract

**Simple Summary:**

To obtain more female individuals in the breeding population of *R. dybowskii*, it is important to explore the sex determination mechanism based on whole genome analysis. In this study, we analyzed the genome survey of male individuals of *R. dybowskii* using next-generation sequencing technology. The estimated genome size of *R. dybowskii* was approximately 3585.05 M. The total size of contigs and scaffolds was 3,748,543,415 and 3,765,862,278 bp, respectively. The N50 length of contigs and scaffolds was 31,988 and 336,385,783, respectively. The *Dmrt1* gene with an 18,893 bp length was obtained from the scaffolding genomes of *R. dybowskii*. Sequence alignment to *Dmrt1* revealed that the two male-specific DNA markers (MSM-222 and MSM-261) were not located on *Dmrt1*. This indicated that *Dmrt1* may not be the only key gene for sex determination in *R. dybowskii*.

**Abstract:**

*Rana dybowskii* is one of the important aquaculture species in Northeast China. The fallopian tubes of female *R. dybowskii* are used to prepare oviductus ranae (an important traditional Chinese medicine). Therefore, *R. dybowskii* females have higher economical value than males. An increasing female *R. dybowskii* population can increase the benefits from *R. dybowskii* culture. However, the genome of amphibians is complex, making it difficult to investigate their sex determination mechanism. In this study, we analyzed the genome of male *R. dybowskii* using next-generation sequencing technology. A total of 200,046,452,400 bp of clean data were obtained, and the K-mer analysis indicated that the depth was 50×. The genome size of *R. dybowskii* was approximately 3585.05 M, with a heterozygosity rate, repeat sequence ratio, and genome GC content of 1.15%, 68.96%, and approximately 43.0%, respectively. In total, 270,785 contigs and 498 scaffolds were generated. The size of the contigs and scaffolds was 3,748,543,415 and 3,765,862,278 bp, respectively, with the N50 length of 31,988 and 336,385,783. The longest contig and scaffold were of the size 137,967,485 and 1,808,367,828 bp, respectively. The number of contigs and scaffolds > 10K nt was 99,620 and 451, respectively. Through annotation, 40,913 genes were obtained, including 156,609 CDS (i.e., 3.83 CDS per gene). Sequence alignment was performed with the assembled scaffolding genome in this study. Two and one fragment had high homology with two male-specific DNA molecular markers of *R. dybowskii* discovered previously (namely, MSM-222 and MSM-261, respectively). In addition, the *Dmrt1* gene of *R. dybowskii* was obtained with a length of 18,893 bp by comparison and splicing. The forward primers amplifying MSM-222 and MSM-261 were located at 322–343 and 14,501–14,526 bp of *Dmrt1*, respectively. However, sequence alignment revealed that MSM-222 and MSM-261 were not located on *Dmrt1*, and only some homologous parts were observed. This indicated that in addition to *Dmrt1*, other important genes may play a crucial role in the sex determination mechanism of *R. dybowskii*. Our study provided a foundation for the subsequent high-quality genome construction and provided important genomic resources for future studies on *R. dybowskii*.

## 1. Introduction

Amphibians form an important group of vertebrates, playing a significant role in the evolution of vertebrates [1]. Amphibians from a transitional group between marine and terrestrial organisms. In the evolutionary history of vertebrates, the transition from aquatic to terrestrial habitats was a huge leap [2]. Before the emergence of amphibians, all vertebrates lived in water. The living conditions on land were much more complex and diverse than those in water. Therefore, amphibians evolved toward higher and more varied and complex directions [3]. Moreover, the larvae of most amphibians live in water, appearing like fish with tails, and breathe through gills. However, the adults live on land and breathe through lungs, indicating their metamorphosis [4]. In such a complex evolutionary position, it was a challenge for the amphibian genome to evolve and help in adapting to such an environment [5,6].

Compared with the genomes of mammals, birds, and fishes, our understanding of amphibian genomes is not sufficient [7]. Currently, AmphibiaWeb (https://amphibiaweb.org/index.html, accessed on 14 January 2024) includes 8717 amphibian species. Although amphibian sex chromosomes have rich diversity [8], only 95 amphibian genomes have been studied, and only 28 of them have been annotated to the chromosome level according to the information provided by the NCBI GenBank database (https://www.ncbi.nlm.nih.gov/genome/?term=amphibians, accessed on 14 January 2024). Many amphibians do not exhibit a high degree of differentiation in sexual chromosomes and have homotypic chromosomes [9,10]. Some studies reported that different amphibians have XY- and ZW-type sex chromosomes [8,11]. More interestingly, XY- and ZW-type sex chromosomes also appear in different geographic populations of the same amphibian species [12].

*Rana dybowskii* is an amphibian belonging to the family Ranidae and the genus *Rana*, which is widely distributed in Northeast China [13]. *R. dybowskii* generally lives on hills near the seaside and mountainous areas up to 1500 m above sea level. They require rich vegetation growing in a humid climate. *R. dybowskii* adults live in water only during winter and breeding seasons. After breeding, they enter the forest after a short period of reproductive dormancy [14,15]. *R. dybowskii* has important ecological and economic value [16]. As an important component of forest ecosystems, *R. dybowskii* feeds on various insects and is also a food source for various birds and small mammals [17]. Second, the fallopian tubes of female *R. dybowskii* are used to prepare a precious traditional Chinese medicine: oviductus ranae [18]. Increasing the female population of *R. dybowskii* can help in maximizing the benefits from its culture. For this, understanding the sex determination mechanism is necessary. However, the genome of *R. dybowskii* has not been thoroughly explored, and only its mitochondrial genome has been assembled (accession number in NCBI: NC-023528). Regarding sex determination, the heteromorphic sex chromosomes of *R. dybowskii* from different places in the Northeast China have not been observed using cytometric methods such as chromosomal karyotype analysis and Ag-band [19]. Xu et al. [20] used DNA molecular marker technology to screen male-specific DNA markers of *R. dybowskii* and reported that the sex chromosome of *R. dybowskii* is of the XY-type. However, further exploration of the genome is needed to reveal the sex determination mechanism of *R. dybowskii*.

Considering the extreme lack of genomic information of *R. dybowskii* and research on the sex determination mechanism of *R. dybowskii*, in this study, we analyzed the genome of *R. dybowskii,* preliminarily exploring the sex determination mechanism. Using DNA molecular marker technology and phenotypic analysis, adult male individuals of *R. dybowskii* were selected, and the genome was studied using the high-throughput sequencing technology. The purpose of this study was to provide a reference for the subsequent sequencing and construction of the chromosome-level genome of *R. dybowskii* and to provide foundational data for studying the sex determination mechanism of *R. dybowskii*.

## 2. Materials and Methods

### 2.1. Phenotypic Sex Determination and DNA Extraction from Tissue Samples

**Figure 1:**
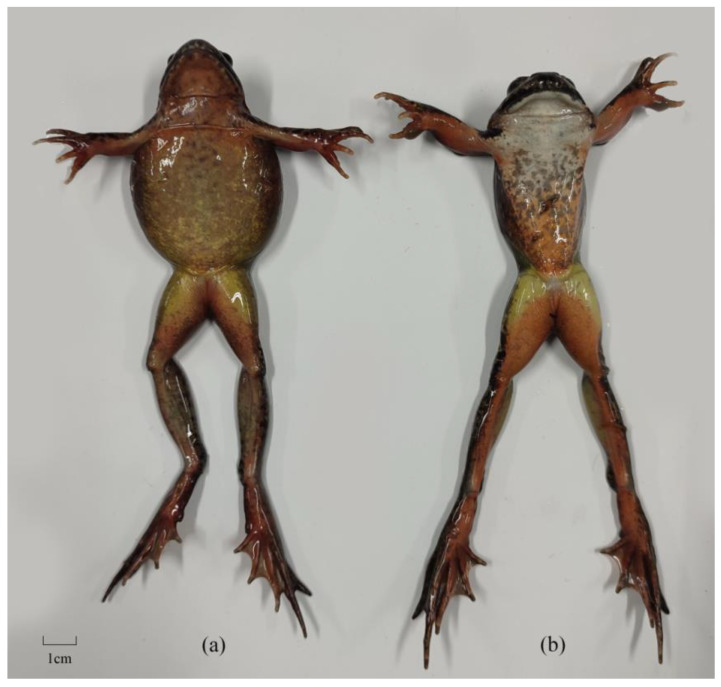
Morphological characteristics of *Rana dybowskii.* (**a**) Phenotypic female of *R. dybowskii*; (**b**) Phenotypic male of *R. dybowskii*.

In total, 12 adult *R. dybowskii* individuals were collected from Taoshan Forestry Bureau in Yichun City, Heilongjiang Province, including 6 male and 6 female individuals (phenotypic sex was identified as indicated in Figure 1). The phenotypic sex of *R. dybowskii* could be identified from external morphological characteristics, which were significantly different between adult males and females. Similar to many frog species, the adult males of *R. dybowskii* had a noticeable nuptial pad at the thumb joint of forelimbs. In autumn and winter, the abdominal fullness of adult females of *R. dybowskii* was higher than that of adult males. After anesthetizing, the thigh muscles of *R. dybowskii* were cut, soaked in 75% ethanol, and frozen at −80 °C. High-quality genomic DNA was extracted from the muscle using the phenol–chloroform method. The quality of extracted DNA was determined using 0.8% agarose gel electrophoresis. The quantity of extracted DNA was determined using a NanoDrop 2000 spectrophotometer (NanoDrop Technologies, Wilmington, DE, USA) and Qubit dsDNA HS Assay Kit on a Qubit 3.0 Fluorometer (Life Technologies, Carlsbad, CA, USA).

### 2.2. Identification of Genotypic Sex

Sex reversal is observed in most amphibians. *R. dybowskii* exhibits sex reversal from genotypic females to phenotypic males [20]. The genotypic male carries the Y chromosome, and its genomic information can better represent all the genomic information of *R. dybowskii*. To ensure that the individuals used for the genome survey were genotypic male individuals, and not pseudo-male individuals with sex reversal, the genotypic sex of collected *R. dybowskii* individuals was determined.

In our previous study, we identified two male-specific DNA markers in *R. dybowskii*, namely, MSM-222 and MSM-261 (lengths of 222 and 261 bp, respectively) [20]. The genotypic sex of *R. dybowskii* was determined as described in our previous study [20]. PCR was performed using a reaction mixture of 20 µL containing 2 µL 10× Taq Buffer, 0.4 µL of 10 mM dNTPs, 1.0 U DNA polymerase (Vazyme, Nanjing, China), 0.8 µL forward primer (10 pm/µL), 0.8 µL reverse primer (10 pm/µL), 2.5 µL of extracted DNA (50 ng/µL), and ddH_2_O to make the volume 20 µL. The primers used are shown in Table 1. PCR was conducted as follows: 5 min of Taq polymerase activation at 95 °C, 5 cycles of denaturation at 94 °C for 1 min, annealing at 35 °C for 1 min, and elongation at 72 °C for 1 min; 35 cycles of denaturation at 94 °C for 1 min, annealing at 51 °C for 1 min and elongation at 72 °C for 1 min, and a final elongation of 7 min at 72 °C. The PCR products were analyzed using 14% polyacrylamide gel electrophoresis.

### 2.3. Library Construction and Sequencing

In total, 1 μg DNA from a male individual was used as the input material, and sequencing libraries were generated using the VAHTS Universal DNA Library Prep Kit for MGI (Vazyme, Nanjing, China), as per the manufacturer’s instructions. Index codes were added to attribute sequences to each sample. Library quantification and size measurements were performed using a Qubit 3.0 Fluorometer (Life Technologies, Carlsbad, CA, USA) and Bioanalyzer 2100 system (Agilent Technologies, Santa Clara, CA, USA). Subsequently, sequencing was performed on a DNBSEQ-T7 platform.

### 2.4. Quality Control

The short-reads from the Illumina platform were quality-filtered by SOAPnuke v 1.3.0 using the following method [21]. First, the adaptors were removed from the sequencing reads. Second, read pairs were excluded if any one end had an average quality < 20. Third, the ends of the reads were trimmed if the average quality was <20 in the sliding window size of 5 bp. Finally, read pairs with any end shorter than 75 bp were removed. The quality-filtered reads were used for genome size estimation.

### 2.5. NT Comparison and K-Mer Analysis

Overall, 10,000 pairs of read data were randomly selected from the filtered high-quality data. Using Blast software v 2.7.1, the sequences were compared with those in the NCBI nucleotide database (NT library), and the top five species with the highest number of comparisons were obtained [22]. To estimate the genome size, heterozygosity rate, and repeat sequence information, the 17-mer distribution of sequencing reads was generated from short libraries using the k-mer method and GCE (version) [23].

### 2.6. Sequence Assembly and Gene Prediction

The assembly software ABySS v 2.0 was used to preliminarily assemble the second-generation data, align the reads to the assembled genome sequence, and summarize the GC bias and repeat sequence of the genome by statistically analyzing the GC content and read coverage depth of the assembled sequence [24]. Finally, the assembly results were assessed using BUSCO software v 5.2.2 [25]. Platanus v1.2.4 was used to concatenate the clean data. The parameters for scaffolding were -t 120 -s 23 -v 23 -l 2 -u 0.2. The parameters for gap closing were -s 23, -k 23, -vo 23, -vd 23, -ed 0.1, -ro 0.6, and -rs 0.8. GlimmerHMM software (v.3.01) was used for the de novo prediction of genes using default parameters. Next, the predicted genes were used for BLAST analysis on the NR and SwissProt databases using BLASTx (DIAMOND v0.8.36 software) (E-value < 1 × 10^−5^).

### 2.7. Searching the Known Male-Specific DNA Markers in the Scaffolding Genome of R. dybowskii

Bowtie2 v2.3.2 (http://bowtie-bio.sourceforge.net/index.shtml, accessed on 13 January 2024) was used for sequence alignment to analyze the positions of the two male-specific DNA markers (MSM-222 and MSM-261) on the genome of *R. dybowskii*. Based on the sequence alignment results, three pairs of primers were designed to verify the accuracy of the localization of two male-specific DNA markers in the assembled genome (Table 2). PCR was performed as described in Section 2.2; the annealing temperature and extension time are shown in Table 3. The PCR product was recovered and sequenced. Further, DNAman was used to identify the upstream and downstream sequences of the two male-specific DNA markers according to the PCR product.

### 2.8. Assembly of the Dmrt1 Gene and Analysis of Its Relationship with Male-Specific DNA Markers

The *Dmrt1* gene of *R. dybowskii* was identified by aligning the *Dmrt1* gene of *Nanorana parkeri* (https://www.ncbi.nlm.nih.gov/nuccore/NW_017306666.1?report=genbank&from=193451&to=252823, accessed on 15 January 2024) to an RNAseq sequence using MMseq software v 0.9.18. and further aligning the *Dmrt1* gene sequence with a matching RNAseq sequence to the assembled genome of *R. dybowskii* [26]. Further, the matching genomic region was extracted. Based on the sequence alignment results and manual correction of intron features (GT-AG principle), the complete *Dmrt1* gene sequence and coding sequence (CDS) were obtained.

According to forward primer sequences for the amplification of the male-specific DNA markers (MSM-222 and MSM-261), the primers were designed (Table 3) for the amplification of the suspected region of the male-specific DNA markers on *Dmrt1*. PCR was performed as described in Section 2.2; the annealing temperature and extension time are shown in Table 3. The PCR product was recovered and sequenced. Further, the sequences of PCR products were compared with the two male-specific DNA markers and their homologous regions on the scaffolding genome to determine whether the two DNA markers are located on *Dmrt1*.

## 3. Results

### 3.1. Identification of the Genotypic Sex of R. dybowskii Individuals

**Figure 2:**
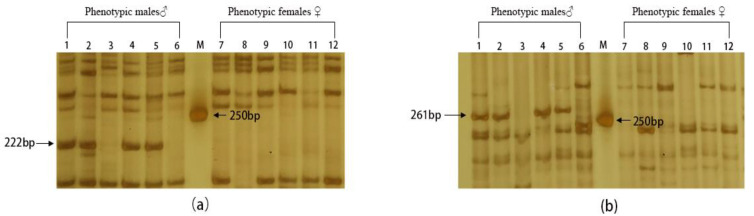
Polyacrylamide gel electrophoresis images for the identification of genotypic males of *R. dybowskii*. (**a**) Amplification results of a male-specific molecular marker (222 bp) with primer Dmrt1-1/EM4; (**b**) Amplification results of a male-specific molecular marker (261 bp) with primer Dmrt1-2/EM7.

The genotypic sex of *R. dybowskii* individuals was identified using male-specific DNA markers of *R. dybowskii*. Among the individuals with a male phenotype, the 222- and 261-bp male-specific DNA markers were amplified in individuals 1, 2, 4, and 5, indicating that these four individuals were males (Figure 2). These male-specific DNA markers were not amplified in individuals 3 and 6, indicating that they were pseudo-male individuals. Meanwhile, the individuals with a female phenotype did not exhibit amplification of the two male-specific DNA markers.

### 3.2. Sequencing Data Statistics and Quality Evaluation

From one *R. dybowskii* sample, a 300–500 bp library was constructed, and 208,387,930,200 bp of raw data were obtained. After filtering and correction, the clean data obtained from the sequencing were of 200,046,452,400 bp. Q20 (%) and Q30 (%) were ≥96.8% and ≥89.7%, respectively (Table 4). The sequencing quality and sequencing error rate were normal (Supplementary Figures S1–S3).

### 3.3. NT Comparison Statistics

The NT comparison results indicated that our sample library was aligned with the DNA of species closely related to *R. dybowskii*. The top five species with the highest matching degree were *R. chensinensis*, *N. parkeri*, *R. rugosa*, *Coregonus* sp., and *R. temporaria*, with the matching rates of 1.95%, 0.835%, 0.76%, 0.61%, and 0.555%, respectively (Table 5). This demonstrated that the library data did not contain significant exogenous contamination.

### 3.4. Genome Size, Heterozygosity Rate, and Repeat Sequence Ratio

**Figure 3:**
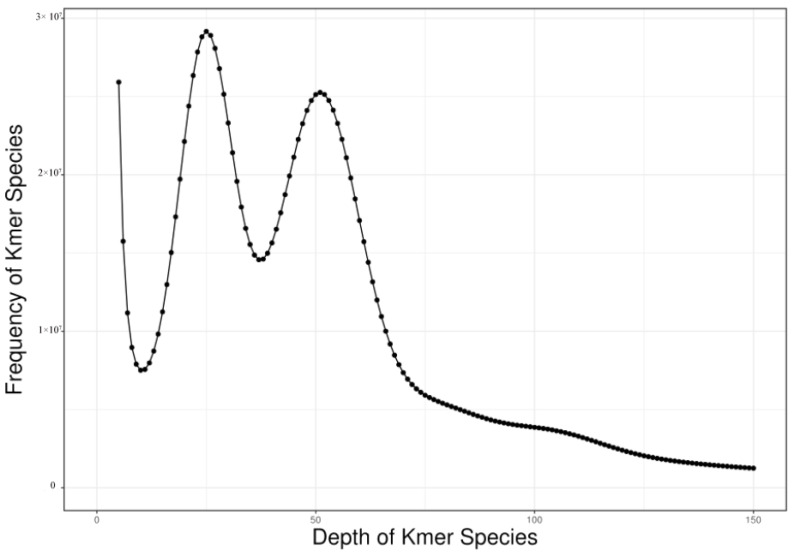
Distribution map of K-mer depth.

The K-mer analysis indicated that the depth of *R. dybowskii* was 50× (Figure 3). The genome size of *R. dybowskii* was approximately 3585.05 M, with a heterozygosity rate of 1.15%, a repeat sequence ratio of 68.96%, and a genome GC content of 1.15%, 68.96%, and approximately 43.0%, respectively. The genome size of *R. dybowskii* was at a moderate level among amphibians with known genomes (Table 6).

### 3.5. Preliminary Scaffolding Genome and Gene Prediction

After splicing and assembly with Platanus v1.2.4, 270,785 contigs and 498 scaffolds were generated. The total size of the contigs and scaffolds was 374,8543,415 and 3,765,862,278 bp, respectively (Table 7). Among 270,785 contigs, 270,745 were confirmed in scaffolds, and 40 were not. The longest contig and scaffold were of 137,967,485 and 1,808,367,828 bp, respectively. The number of contigs and scaffolds > 10K nt was 99,620 and 451, respectively. In this study, the N50 length of contigs and scaffolds was 31,988 and 336,385,783, respectively, and the L50 length was 29,092 and 2, respectively. Through annotation, 40,913 genes were obtained, including 156,609 CDS (i.e., 3.83 CDS per gene). The length of the CDS was 52,923,470, and the CDS length per gene was 1294 bp.

### 3.6. Homologous Sequences of Male-Specific DNA Markers in the Scaffolding Genome

**Figure 4:**
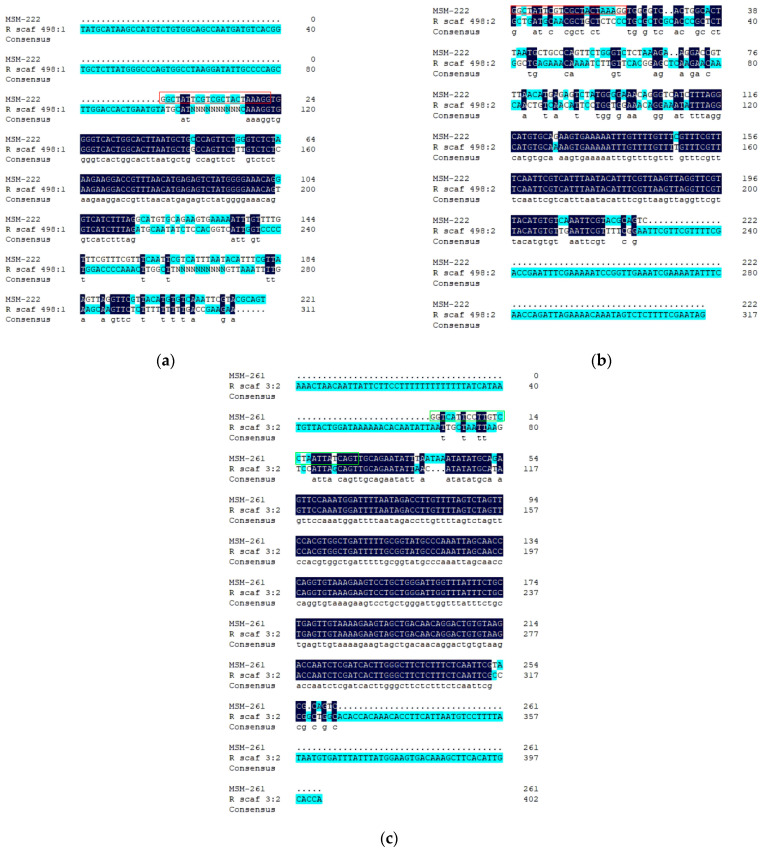
Homologous sequences of MSM-222 and MSM-261 in the scaffolding genome of *R. dybowskii*. (**a**) Comparison between MSM-222 and RanDyc_scaf_498:645910742-645911053 (R scaf 498:1); the sequence shown in the red box is of the primer Forward1; (**b**) comparison between MSM-222 and RanDyb_scaf_498:505645920-505646238 (R scaf 498:1); the sequence shown in the red box is of the primer Forward1; (**c**) comparison between MSM-261 and RanDyb_scaf_3:159744804-159745236 (R scaf 3:2); the sequence shown in the green box is of the primer Forward2.

After comparing with our assembled scaffolding genome, two fragments (>RanDyb_scaf_498:645910742-645911053 and RanDyb_scaf_498:505645920-505646238) exhibited high homology to MSM-222, and one fragment (>RanDyb_scaf_3:159744804-159745236) exhibited high homology to MSM-261 (Figure 4).

**Figure 5:**
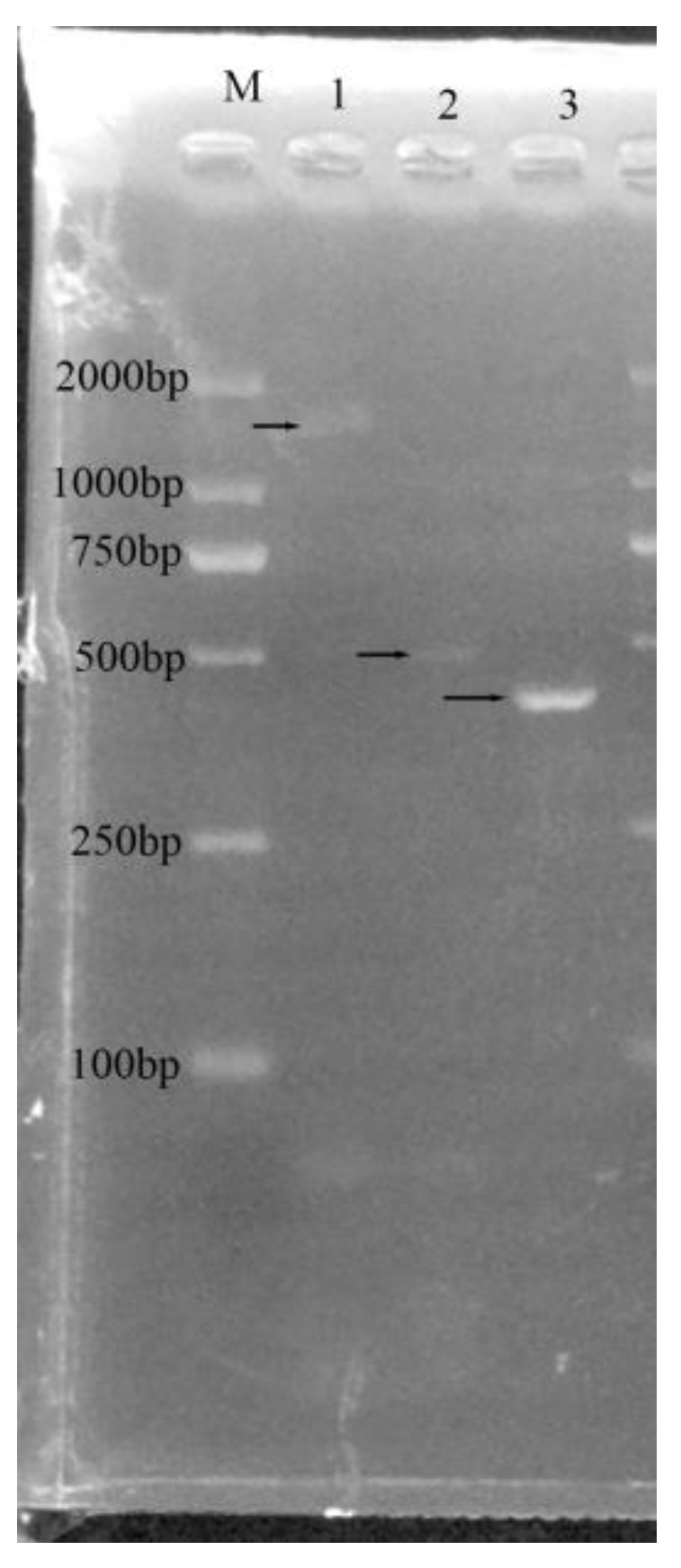
Agarose gel electrophoresis of three pairs of primers for the amplification of scaffolds. M Lane: Marker; Lane 1: PCR products of 289F/289R; Lane 2: PCR products of 303F/303R; Lane 3: PCR products of 403F/403R.

**Figure 6:**
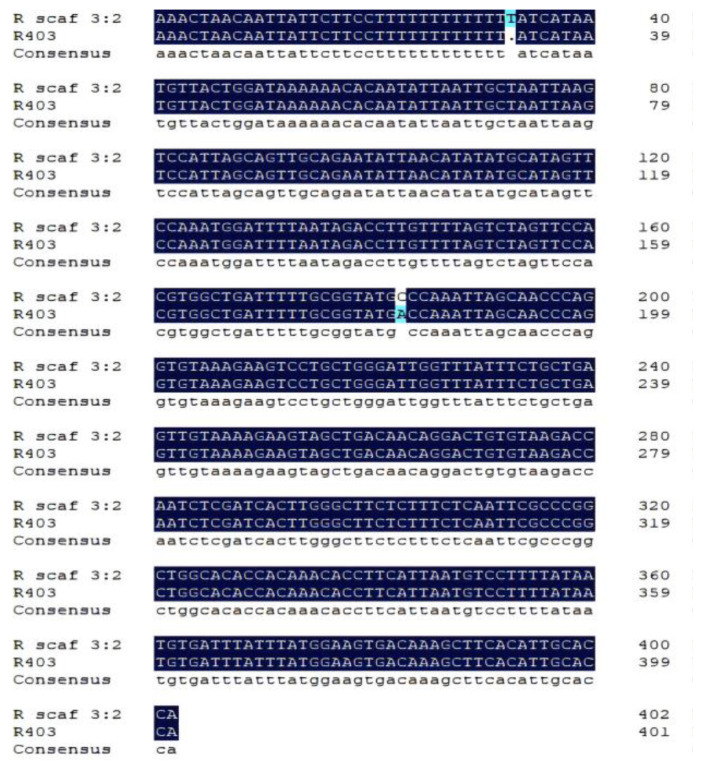
Comparison between R scaf 3:2 and R403.

**Figure 7:**
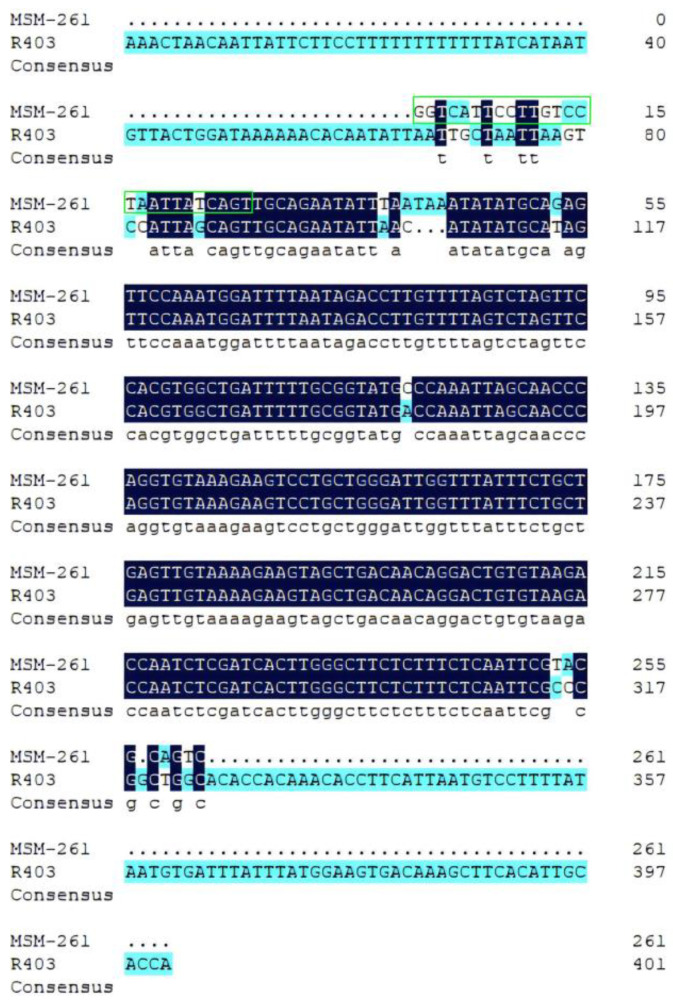
Comparison between MSM-261 and R403. The sequence shown in the green box is of the primer Forward2.

Based on the three sequences on the scaffolding genome, three pairs of primers were designed to verify their authenticity (Table 2). Their homology was compared with MSM-222 and MSM-261. The amplified products of the primer pairs 289F/289R and 303F/303R were blurry bands of approximately 1700 and 500 bp in length, respectively, and the amplified product of primer pair 403F/403R was a bright band of approximately 400 bp in length (Figure 5). They were named R1700, R500, and R403, respectively. After recovering and sequencing the PCR products, the sequence of only R403 was obtained, which was highly similar to that of >RanDyb_scaf_3:159744804-159745236 (Figure 6). By comparing R403 with the corresponding male-specific DNA marker (MSM-261), it was observed that R403 contained the double-ended sequence of MSM-261 (Figure 7).

### 3.7. The Dmrt1 Gene Sequence of R. dybowskii and Its Relationship with Male-Specific DNA Markers

Via comparing and splicing, the *Dmrt1* gene of *R. dybowskii* with a length of 18,893 bp was obtained. The Forward1 (Dmrt1-1: primer for amplifying MSM-222) was located at 322–343 bp of *Dmrt1*, and the Forward2 (Dmrt1-2: primer for amplifying MSM-261) was located at 14,501–14,526 bp of *Dmrt1*.

**Figure 8:**
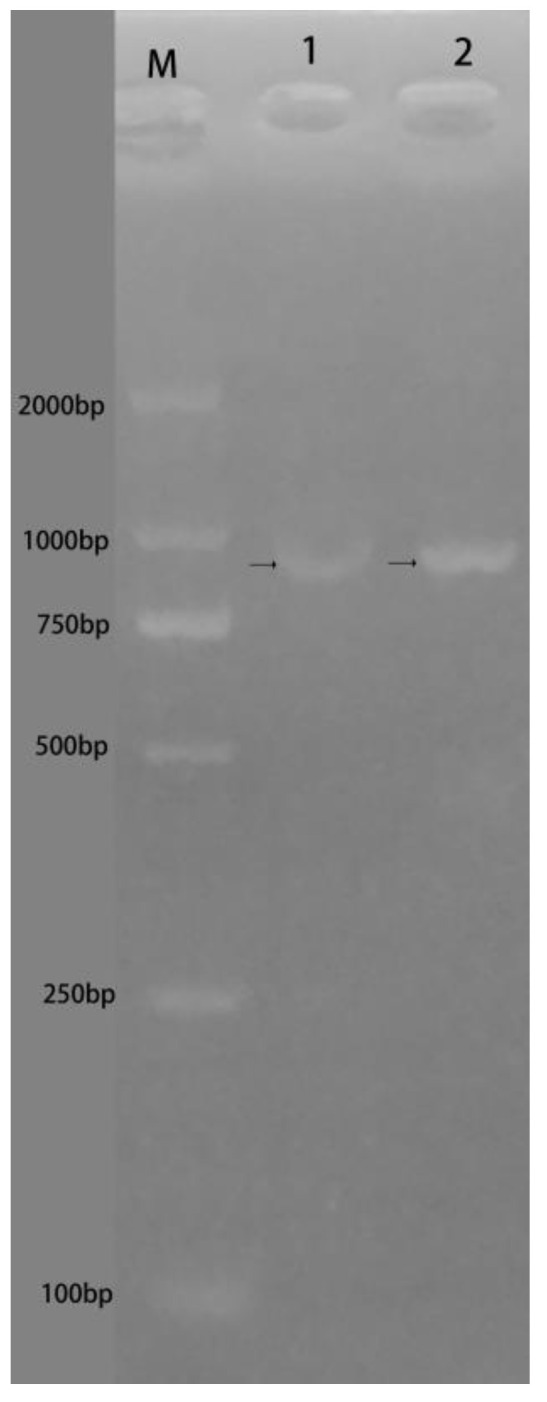
Agarose gel electrophoresis of three pairs of primers for amplifying the partial sequence in *Dmrt1*. M Lane: Marker; Lane 1: PCR products of 913F/1021R; Lane 2: PCR products of 940F/940R.

Two pairs of primers 913F/1021R and 940F/940R, respectively, were designed for amplifying the 1–1020 and 13,828–14,767 bp sequences of the Dmrt1 gene of *R. dybowskii*, including Forward1 (Dmrt1-1) and Forward2 (Dmrt1-2). The amplified products shown in Figure 8 were recovered by agarose gel electrophoresis and sequenced, obtaining 1092 bp (including Forward1 Dmrt1-1) and 940 bp (including Forward2 Dmrt1-2) sequences.

**Figure 9:**
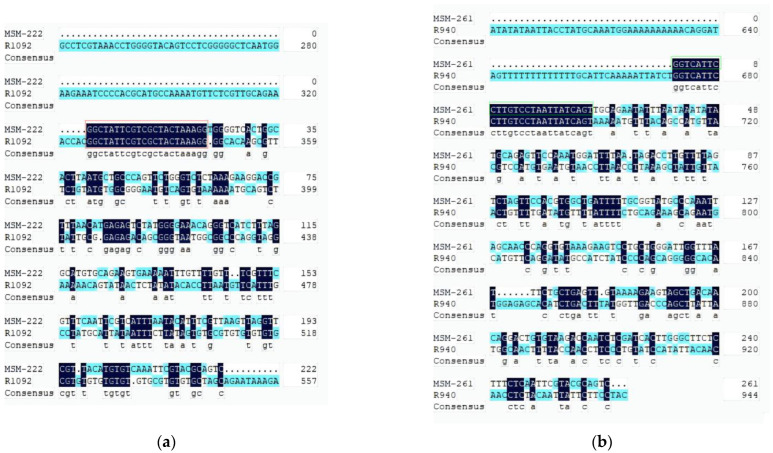
Homologous sequences of MSM-222 and MSM-261 in *Dmrt1* of *R. dybowskii*. (**a**) Comparison between MSM-222 and R1092; the sequence shown in the red box is of the primer Forward1; (**b**) comparison between MSM-261 and R940; the sequence shown in the green box is of the primer Forward2.

The sequence of MSM-222 was compared with the 1092 bp fragment, and that of MSM-261 was compared with 940 bp fragments. It was observed that MSM-222 and MSM-261 were not located on *Dmrt1*, although some homologous parts were observed (Figure 9).

## 4. Discussion

From early Sanger sequencing to next-generation sequencing (NGS) technology and further to third-generation single-molecule sequencing technology, DNA sequencing technology has been driving the development of life science research [27,28]. A genome survey based on NGS is an effective means of evaluating the complexity of the genome. It is necessary to determine the genomic characteristics before assembling the third-generation sequencing of the genome [29,30]. Due to the direct impact of the genome size and heterozygosity on sequencing strategies, cycles, and other factors, low-depth NGS data based on small-fragment libraries are used to evaluate the size and complexity of the genome through bioinformatic methods [31,32]. In our study, the genome size of *R. dybowskii* was observed to be approximately 3585.05 M, with a heterozygosity rate, repeat sequence rate, and genome GC content of 1.15%, 68.96%, and approximately 43.0%, respectively. This study provided guidance for the development of subsequent strategies for whole genome de novo sequencing and assembly for this species.

The results of NT comparison analysis on NCBI indicated that the similarity between *R. dybowskii* and *R. chensiniensis* was the highest, indicating that *R. dybowskii* and *R. chensiniensis* had the closest genetic relationship. For a long time, the taxonomic status of *R. dybowskii* had been relatively chaotic and unclear [14]. From the morphometrics and external characteristics, *R. dybowskii* and *R. chensinensis* are very similar; therefore, *R. dybowskii* was considered a subspecies of *R. chensinensis* for some time [33]. However, after in-depth analysis, researchers observed slight differences in the body size and type of the seventh pair of chromosomes between *R. dybowskii* and *R. chensinensis* [14]. In our study, an analysis of the genome of *R. dybowskii* via NT data library comparison provided a theoretical basis for its taxonomical analysis.

Overall, 28 annotated amphibian genomes at the chromosome level were included in the NCBI GenBank (https://www.ncbi.nlm.nih.gov/genome/?term=amphibians, accessed on 14 January 2024) (the smallest genome: *Spea bombifrons* 0.96 G and the largest genome: *Ambystoma* mexicanum 28.20 G; Table 7). In our study, the genome of *R. dybowskii* was observed to be approximately 3.5 G, whereas that of *R. temporaria* (also belonging to the frog genus) is 4.1 G. In the NT comparison on NCBI, the genetic relationship between *R. dybowskii* and *R. temporaria* was relatively close. However, karyotype analysis revealed that the chromosome number of *R. dybowskii* and *R. temporaria* was 24 and 26, respectively [16,34]. At present, the entire genome of *R. temporaria* has been annotated at the large chromosome level [35]. To further analyze the genetic differences between *R. dybowskii* and *R. temporaria*, as well as the sex determination mechanism of *R. dybowskii*, the entire genome of *R. dybowskii* should be sequenced based on a genome analysis.

Genome sequencing is of significant help in revealing the mechanisms of sex determination [36,37]. The research on the sex determination mechanism of *R. dybowskii* is still at the preliminary stage. At the cellular level, heterosexual chromosomes were not observed in *R. dybowskii* [16]. At the molecular biology level, two male-specific DNA molecular markers of *R. dybowskii* were screened using target region amplified polymorphism technology, and it was preliminarily confirmed that the sex chromosome of *R. dybowskii* is of XY-type. However, due to the complex and diverse sex determination mechanisms of amphibians [10,38,39], our in-depth research on the sex determination mechanism of *R. dybowskii* has been greatly hindered. Based on the genome survey, in this study, we preliminarily spliced the genome of *R. dybowskii* and selected homologous fragments of male-specific DNA molecular markers in the scaffolding genome. This provided new clues for the further exploration of sex determination mechanisms in *R. dybowskii*. Meanwhile, the forward primers for amplifying the male-specific DNA molecular markers of *R. dybowskii* were anchored to *Dmrt1* [19]. In addition, Dmrt1 is a differentially expressed gene in the testes of male and pseudo-male individuals [16]. Therefore, we previously speculated that the sequence of the male-specific DNA molecular marker of *R. dybowskii* would be present on Dmrt1 [19]. However, after comparing the male-specific DNA molecular marker sequences and *Dmrt1* sequence, where the forward primers were located, we observed that these two molecular markers were not present on *Dmrt1*. They only shared the sequences of forward primers that led to the amplification of the two male-specific DNA molecular markers in PCR. Thus, the sex determination mechanism of *R. dybowskii* was observed to be very complex, not as simple as we previously thought [19]. In addition to *Dmrt1*, other key genes may play a role in sex determination in *R. dybowskii*.

## 5. Conclusions

In this study, the genome survey of male *R. dybowskii* was analyzed, which provided a foundation for the subsequent high-quality genome construction and important genomic resources for the research on the *R. dybowskii*. The genome size of male *R. dybowskii* was observed to be approximately 3585.05 M. Genomic comparative analysis revealed that *R. dybowskii* has the closest genetic relationship with *R. chensinensis*. The two male-specific DNA molecular markers of *R. dybowskii* (MSM-222 and MSM-261) were not present on *Dmrt1,* and their exact locations in the genome are still unknown, which should be explored in future studies. These two markers and *Dmrt1* only shared the sequences of forward primers that amplified the two male-specific DNA molecular markers in PCR. In conclusion, further genomic research is needed to understand the sex determination mechanism of *R. dybowskii*.

## Figures and Tables

**Table 1:** Primer sequences and pairs of primers used for the identification of the genotypic sex of *Rana dybowskii*.

Marker	Primer		Sequence (5′→3′)
MSM-222	Forward1	Dmrt1-1	GGCTATTCGTCGCTACTAAAGG
	Reverse1	EM4	GACTGCGTACGAATTTGA
MSM-261	Forward2	Dmrt1-2	GGTCATTCCTTGTCCTAATTATCAGT
	Reverse2	EM7	GACTGCGTACGAATTGAG

**Table 2:** Primer sequences and primer pairs used for identifying the location of two male-specific DNA markers (MSM-222 and MSM-261) on the assembled genome of *R. dybowskii*.

Verify Target	Primer	Sequence (5′→3′)	Annealing Temperature (°C)	Time of Extension (s)	Position of Template
Upstream MSM-222 in scaffolding genomes	289F	TGCATAAGCCATGTCTGTG	51.5	60	>RanDyb_scaf_498:645910742-645911053
	289R	AGAACTTGCTTCAAAATTTAA			
Downstream MSM-222 in scaffolding genomes	303F	TGATGCAACGCTGCTCTC	47.5	60	RanDyb_scaf_498:505645920-505646238
	303R	CTATTTGTTTTCTAATCTGGTTG			
Double-ended MSM-261 in scaffolding genomes	403F	AAACTAACAATTATTCTTCC	51	60	>RanDyb_scaf_3:159744804-159745236
	403R	GTGGTGCAATGTGAAGC			

**Table 3:** Primer sequences and primer pairs used for identifying the suspected location of two male-specific DNA markers (MSM-222 and MSM-261) on the *Dmrt1* gene of *R. dybowskii*.

Verify Target	Primer	Sequence (5′→3′)	Annealing Temperature (°C)	Time of Extension (s)	Position of Template
Suspicious location of MSM-222 in Dmrt1	913F	TTGTGGAGGATTGTTACCCAAT	61.4	90	>RanDyb_Dmrt1:1-1020
	1021R	CTCTCCACCCAAAACAAAAATAAT			
Suspicious location of MSM-261 in Dmrt1	940F	AAACTAACAATTATTCTTCC	47.8	60	>RanDyb_Dmrt1:13828-14767
	940R	GTGGTGCAATGTGAAGC			

**Table 4:** Quality assessment of the sequencing data.

Data Type	ReadNum	BaseCount (bp)	ReadLength (bp)	Q20 (%)	Q30 (%)	GC Content (%)
raw data	1,389,252,868	208,387,930,200	150;150	99.0;96.8	95.2;89.9	43.2;43.1
clean data	1,333,643,016	200,046,452,400	150;150	99.0;96.8	95.2;89.7	43.1;43.0

Note: Q20, the ratio of data with an accuracy > 99% in total data. Q30, the ratio of data with an accuracy > 99.9% in total data.

**Table 5:** Result of NT library comparison.

Species	Reads 1	Reads 2	Total (%)
*Rana chensinensis*	201	189	1.95
*Nanorana parkeri*	91	76	0.835
*Rana rugosa*	83	69	0.76
*Coregonus* sp.	65	57	0.61
*Rana temporaria*	54	57	0.555

**Table 6:** Comparison of the genome size of amphibians.

Species	Genome Size (G)
*Spea bombifrons*	0.96
*Xenopus tropicalis*	1.5
*Pyxicephalus adspersus*	1.6
*Leptodactylus fuscus*	2.2
*Engystomops pustulosus*	2.6
*Xenopus borealis*	2.7
*Xenopus laevis*	2.7
*Eleutherodactylus coqui*	2.8
*Pelobates cultripes*	3.1
*Hymenochirus boettgeri*	3.2
*Leptobrachium ailaonicum*	3.5
*Leptobrachium leishanense*	3.5
*Rana dybowskii*	3.5
*Monodelphis domestica*	3.6
*Geotrypetes seraphini*	3.8
*Bufotes viridis*	3.8
*Discoglossus pictus*	3.9
*Hyla sarda*	4.1
*Rana temporaria*	4.1
*Gastrophryne carolinensis*	4.3
*Bufo gargarizans*	4.5
*Microcaecilia unicolor*	4.7
*Bufo bufo*	5
*Rhinatrema bivittatum*	5.3
*Ranitomeya imitator*	6
*Pseudophryne corroboree*	8.9
*Bombina bombina*	10
*Ichthyophis bannanicus*	12.4
*Ambystoma mexicanum*	28.2

**Table 7:** Preliminary scaffolding genome of *R. dybowskii*.

Item	Contig	Scaffold
Total Number	270,785	498
Total size contig/scaffolds (bp)	374,8543,415	3765,862,278
Longest contig/scaffolds (bp)	137,967,485	1,808,367,828
Shortest contig/scaffolds (bp)	62	881
Number of contig/scaffolds > 1K nt	233,279	497
Percentage of contig/scaffolds > 1K nt	86.10	99.80
Number of contig/scaffolds > 10K nt	99,620	451
Percentage of contig/scaffolds > 10K nt	36.80	90.60
Number of contig/scaffolds > 100K nt	2274	213
Percentage of contig/scaffolds > 100K nt	0.80	42.80
Number of contig/scaffolds > 1M nt	13	41
Percentage of contig/scaffolds > 1M nt	0.00	8.20
Number of contig/scaffolds > 10M nt	2	14
Percentage of contig/scaffolds > 10M nt	0.00	2.80
Mean contig/scaffolds size	13,843	7,561,972
Median contig/scaffolds size	5837	69,683
N50 contig/scaffolds length	31,988	336,385,783
L50 contig/scaffolds count	29,092	2
contig/scaffolds %A	28.2	28.07
contig/scaffolds %C	21.13	21.03
contig/scaffolds %G	21.15	21.05
contig/scaffolds %T	28.22	28.09
contig/scaffolds %N	1.3	1.75
contig/scaffolds %non-ACGTN	0	0
Number of contig/scaffolds non-ACGTN nt	0	0
Percentage of assembly in scaffolded contigs	100	
Percentage of assembly in unscaffolded contigs	0	
Average number of contigs per scaffold	543.7	
Average length of break (≥25 Ns) between contigs in scaffold	64	
Number of contigs in scaffolds	270,745	
Number of contigs not in scaffolds	40	

## Data Availability

The data presented in this study are available on request.

## Supplementary Materials

The following supporting information can be downloaded at: https://www.mdpi.com/article/10.3390/ani14202968/s1.
