# Supplementary material for: Genome Survey of Male Rana dybowskii to Further Understand the Sex Determination Mechanism
Source: Animals (Basel). 2024 Oct 14;14(20):2968. doi: 10.3390/ani14202968 (PMC11503867; doi:10.3390/ani14202968)
Supplement: Supplementary file 1 [file animals-14-02968-s001.zip › Supplementary Sequence.pdf]

The sequence in our study:

supplementary sequence S1. Sex-specific marker from dmrt1-1/EM4 (222bp)

GGCTATTCGTCGCTACTAAAGGTGGGGTCACTGGCACTTAATGCTGCCCAGTTCTG

GGTCTCTAAAGAAGGACCGTTTAACATGAGAGTCTATGGGGAAACAGGGTCATCT

TTAGGCATGTGCAGAAGTGAAAAATTTGTTTTGTTTCGTTTCGTTTCAATTCGTCAT

TTAATACATTTCGTTAAGTTAGGTTCGTTACATGTGTCAAATTCGTACGCAGTC

supplementary sequence S2. Sex-specific marker from dmrt1-2/EM7 (261bp)

GGTCATTCCTTGTCCTAATTATCAGTTGCAGAATATTTAATAAATATATGCAGAGTTC

CAAATGGATTTTAATAGACCTTGTTTTAGTCTAGTTCCACGTGGCTGATTTTTGCGG

TATGCCCAAATTAGCAACCCAGGTGTAAAGAAGTCCTGCTGGGATTGGTTTATTTC

TGCTGAGTTGTAAAAGAAGTAGCTGACAACAGGACTGTGTAAGACCAATCTCGAT

CACTTGGGCTTCTCTTTCTCAATTCGTACGCAGTC

supplementary sequence S3
>RanDyb_scaf_498:645910742-645911053
TATGCATAAGCCATGTCTGTGGCAGCCAATGATGTCACGGTGCTCTTATGGGCCCA
GTGGCCTAAGGATATTGCCCCAGCTTGGACCACTGAATGTATGCATNNNNNNNNNN
CAAAGGTGGGGTCACTGGCACTTAATGCTGGCCAGTTCTTTGTCTCTCAAGAAGG
ACCGTTTAACATGAGAGTCTATGGGGAAACAGTGTCATCTTTAG------------ATGCAA
TATCTCCACGGTCATTGGTCCCCTGGACCCCAAACTTGGCTTNNNNNNNNNNGTTA
AATTTTGAAGCAAGTTCTCTTTTTT---TTTGACCGAAGAA

supplementary sequence S4
>RanDyb_scaf_498:505645920-505646238
GCTGATGCAACGCTGCTCTCCCTGCGCTCGCACCCGCTCTGGCTGAGAAACAAAA
TCTTGTTCA----CGGAGCTCAAGAACAACAACTGTCAACAT-----TCCTGGTGGAAAC
AGGA-AATATTTAGGCATGTGCAAAAGTGAAAAATTTGTTTTGTTTTGTTTCGTTTC
AATTCGTCATTTAATACATTTCGTTAAGTTAGGTTCGTTACATGTGTTGAATTCGTTT
TCGGAATTCGTTCGTTTTCGACCGAATTTCGAAAAATCCGGTTGAAATCGAAAATA
TTTCAACCAGATTAGAAAACAAATAGTCTCTTTTCGAATAGA

supplementary sequence S5
>RanDyb_scaf_3:159744804-159745236
AAACTAACAATTATTCTTCCTTTTTTTTTTTTTATCATAATGTTACTGGATAAAAAAC
ACAATATTAATTGCTAATTAAGTCCATTAGCAGTTGCAGAATATT---AACATATATGCA
TAGTTCCAAATGGATTTTAATAGACCTTGTTTTAGTCTAGTTCCACGTGGCTGATTT

TTGCGGTATGCCCAAATTAGCAACCCAGGTGTAAAGAAGTCCTGCTGGGATTGGTT
TATTTCTGCTGAGTTGTAAAAGAAGTAGCTGACAACAGGACTGTGTAAGACCAATC
TCGATCACTTGGGCTTCTCTTTCTCAATTCGCCCGGCTGGCACACCACAAACACCT
TCATTAATGTCCTTTTATAATGTGATTTATTTATGGAAGTGACAAAGCTTCACATTGC
ACCAC

supplementary sequence S6

assembly of Dmrt1 gene of *R. dybowskii*

CCACATCTGGCCCCAGGGCCGCAGTTTGGAGACCACTGTCCTATGGGATATTTTTAT
GGCATTTTTATTTATTTAATTTTTTTAATAGTAATGGTGGCGATCAGCGATTCTTAGC
GGGACTGTGACATTGTGGCAGACAAATCAAACACCTAACTGACACTTTTGACACT
TTTTGGAAACTGGTGAATTTATTAGTGAACAGTGCTAAAAATAGAGAAGGGGATAA
CCGCCAGGGGCGATCAAAGAGTTAAGTGCGTCCCTAGGGGGTGCTTGCTAACTGT
GTGGAAGATGGATGAACACAAAGATCTATGTTCGTGTCTATTGATAATCACCAGTC
TGTCTTCAATCATCTCCTTTTTATTACTATTATTTTAATAAATTTTTTATGTTTACATAA
ACTTGGATCACCATTTTGTGGTTATATCCATCTTTCTGGGACTACTAGCATCAGCCA
GCAGTACTACTTGTTCATCTGACTACACAGCTATGTGAATGGGGGGTATCCTCCTCG
CTGTGTCCTTATAGTCTGACAGAGAGGAGACTTGTCTGCCATCAGAATACACAGAT
CAGTCGGGCTGATGAAGCGGAAAATTTCCAACACAGAAGTCAATCAGTAGATCGA
CTTCTGTTCAACCACAATGCCCACGTGGATCGAAATTCGACTGGCCCCTGCTGAAT
TGGCTGAATTTTGATCTATATATGGGCAGCTTAACACACTTTTCATTTCTAGGATGGC
AGCTCTCACTCACAGTATGTACAGTATGTATGGAATAGCAGCATTGTTTCCATAGAA
CACTAGGGTTTATTTACTAAAGGCAAATAGACTGTGCGTTTTGCACGCATTTCCCCC
CATTTTTTGTGAATGTGGTGAAGCTTCGTTTTGTAAAGTATACCCAATCAGGTGAA
AGGAAAAAAACCTGCATTTTTGCTTGCACATGTTTAGATGACGAAAATCAGCAGA
GCCTCACCTCATTCACTAAATAAACTGAAGAAATTACTGCACCTGTAAAGTGTAAA
TTATATTTCCCTTTGGTAAATCACCTGCAAAGTGTGTTAGGACAATAACGTATTTCC
CTCTTCTCCACATCTCCTTAACATAACAGAGAGGTAACTGGGCAGGACTGATAGTA
CTGCTCGGGATAGCACTTCAGGGTGCACTGGGCAAAGGACAACAATTGATTTCTG
GTAGGATCAATGGGTTTTTGTTTGCACCTAGGGAATAGTTAAAACACTTGCAAATT
GGATATTTACATTTTAACAAAGCAAAAGGGAGCAAATATAAGTTTGTTGTTAGGGT
TTACATGCATTTTAACTAGTTCCTCCTGGGCTGTGGCATAATGACGCCAGGGGGAA
CCTCTCGTCGAATCAGGTGGACATCATATGACGTCCTCCTGGATCGCACCCCGCGG
GGCGGCGCAGCGACAGCATCTGTCACCAGTCAATGACTGATCACTGTACTAGCCC
CCTGCCGATACAAAGTGAATGCTATAACCTATCACTGCAGGTCACATGACATTTATA
CTTTTAAGCCATCCATTGTATACAATTGTGTTGGTCAGTGTGATCACATGGTACAAA
CTGGGGCAATCACAGCTCATCTGTACCGTGTGATTAGCTCTGACCAATCACAGCTA
ATCACAACAAATACTGAATCAATCGATTTCATTCAGTAAAATGCTTGCATATAACAA
TTACATTTATTGCTATATGCAAGCATAATGTGTAAAAAATAAATAAAAATACTGATCA
CTTTCGCAGAGTAGTACAATGCTACTTTGGTAACATATTGCCCTGGTCAAAGTGTGT
AAAAAAAACACACACAAACAACCCGTACAAGAAAAAAAAAAAATTTTTTTTTTG
TCATACTGAGTACTGTAACCAGTTTGTGTCCCTGATCACCACCACACCTGTTATATG
ATGCTGTACTACACTGGTGACAGTATGTATAAAAAGGGGTCATTTGGGGGATATTTG
TACTGCCCTGACATTTTTATGCCTCAAGAAATGAGAAATGAGGCCATCAGGATTAG

TAAATTTTCAGATATATACACACCATAGTAGGACTCTATAACTTTCCGACATACTAAA
TAATTTACACTAATTTGGGCTAATTTCAGGAAATGTAGCAGAAAAAATAAAAAAAC
TCTGTGGTGATTAAATACCACCAAAAGAAAGCCATTTGTGTGAAAAAAAATTTTGA
AAATGTAGTTTGGGTACAATGTTGCATGACAATTGTCATACAAAGTGTGACAGCAC
GTAAAGCATGAACTTGGCCTGGGTAGGAAGGGAGTAAAAATGCCCTGTATTGCAG
TGGTTAAAACACTGGCAGTTATTTACTTGATAGGGGCTTCAAGTGTGGCCCCTGAC
ATCACCATAAGATATCATGTATTTTGTAAATGCAATCCTAAAAAAGCTGTCGGACTG
AAGTCATACAACACAAGATGGTCAGACTGTAGACATGCTCTAGGAGTTGCAAAGG
AAAGGAATGTACAAACAGGAAAAAGTAAGGCCGCGTACACACGATCGGTCAAAA
CCAATGAAAACGGACTGAAGTTCAGTTTCATCGGTCCAAACCGATCGCGTGTAGG
CCCCATCGGTCAGTTGTCCTTCGGTCCAAAAACAGAGAACTTGCTTTAAAATTGAA
CCAATGGACGCCTGAGCGATGGGTCAAAACCAATCGTTAGTACGCAAAGGTATCA
GTCAAAAGGCCGCACAAGCTCAGAATCAAGTCGACGCATGCTTGGAAGCAGTGA
ACTTGCCCATCCATCCCTGCCCAATACTCTGCAATACCCTGTGCAATACTCCAATAC
CCTGTGCAATACCCTGCAATACACCGGAATACTCCAATACCCTGTGCAATACTCTGA
AATACCCCAATACTCTGCAATACTCCAATACCATGCAATATTGCAATACTCCACAAT
ACGCCCCCCCCCTCCCCTCCCGCCACCCTCCGATGCTTTTACAGACTCACCGCTAC
GATCAAATCCAGGATTGTTTTTTTATTGCAGTCTTCCCAGCCTAGAGGTGAGATGTG
GGGTCTTATTGACCCCACATCTCACTGTAAAGAGGACCTGTCATGCTATATTGCTAT
TACAAGGGATGTTTACATTCCTTATAATAAGAATAAAAGTGATCAAAAAAATATTTT
TGGGGAAAAAAGTGTCAAACTAAAATAAATGAAGTAAAATGAACAATAAATTATTA
TTATTCTTTTTAACGCTCCCCTGTCCCCGTGTGCGCGCATTCAGAAGTGAACGCATA
CATAATTCCCGCCGACATATGAAAACGGTGTTCAAGCTACACATGTGAGGTATCGC
TGTGAACGTTAGAGCGAGAGCAATCATTTTGGCCCTAGACCTCCTCTGTAACTAAA
AACATGTAACCAATAAAAAAATTAAAGCGTCGGATTTTTAACCACTTAAGGACCTT
GGGTGTTTTTCAGATTTGGTGTTTGCAAGACTAAAACTGTTTTTTCTGCTAGAAAAT
AACTTCAAACCCCCAAACATTATATATATTTTTTTCCTACAGAATAAAATAGTGGTCA
TTGCAATACTTTTTATCACACCGTATTTGCGCAGCGGTCTTACAAGCGCACTTTTTT
TTTGAAAAAATTCACTTTTTAATAAATTTGGCCCAATTTTTTTATATATTGTGAAAGA
TAATGTTACGCCGAGTAAAATGATACCCAACATGTCACGCTTAAAAATTGCGCCCG
CTCGTGGCATGGCGTCAAACTTTTACCCTTAAAAATCTCGATAGGCGACGTTTAAA
AAATTCTATAGGTTGCATCTTTTGAGTTACAGAGGAGGTCTAGGGCTAGAATTATTG
CTCTCGCTCTAACGATCGCGGCGATACCTCTCATATGCGGACGAGCTCGTCGGGAC
GGGGCGCTTTGTTTTTTTGTTTTCTTATTT
ATTTTTATTTATTTTATTACTTTTTACACTGAAAAAAATAAATAAAAATGTGATCACT
TTTATTCCTATTANNNNNNNNNNNNNNNNNNNNNNNNNNNNNNNNNNNNNNNNNNNN
NNNNNNNNNNNNNNNNNNNNNNNNNNNNNNNNNNNNNNNNNNNNNNNNNNNNNNN
NNNNNNNTCCTCTGTTATGATAGGTTGTAAATCTTTGATTATCTTTTTTATCTCTTCT
AGTGCTGGATTGTATGTAGTGACTAGATTTACATGGATATTTGTTTTTTTCTCTTTTT
GTATTGGAGGAGATTTTCTCTTTGGGTTTTCAAGGCTGTGTTTATGCTGTTGTTGAT
GGTTTGGTCTTTGTAACCTTTCTTATAGAAGGAATATGCTAGTGTTTTGAGATGTTTA
TCTCTGTCTTTTGGGTCTGAACATATTCAGTGGTATCTGATGGATTCTATTGTGTGTA
TCCAGCTTAAAAAGGAGGTTCACACAAAAAGTGAACCTCGGCTTTTTGGATCCCT
CCCCCCTCCGGTGTCACATTTGGCACCTTTCNNNNNNNNNNNNNNNNNNNNNNN

NNNNNNNNNNNNNNNNNNNNNNNNNNNNNGAGTGAGTGAGTGAATGACTTGTATA
GCGCTACTAGTGTGAACTGAATCGCCTCAAGGCGCTTTTTTGCAGCCAGTGTCTTC
CTGGCTGGTACGGTCATTTACCCCGTAGGATCTTGACACACTTCGGACACACAGTC
GAACACACATATATACATATATATATATATGCACCACTTGGGGGTTCTGACTCCCGTG
GGAGTCCAGCCTCGTCACCCGCCAGGAGAAAGCAGAGACCAGTAACGGCGCACC
GCGATCCTTGCGCATGTGCAGTAGGGAATCGGGCAGTGAAGCTGCAAGGCTTCAC
TTCCTGATTCCCTCACCGAGGATTGCGGCGGAAGTAGCTCCTTGGGGGAGCTGGA
CAGGTAAGTGTCCATTTTTTAAAAGTCAGCAGCTGTAGTATTTGTAGCTGCTGGTTT
TAATGCCTTTCTATCATTGTAAACCCTAGAGATGGTTGTGCATGAAAATCCCAGTAT
ATCAGCCGTTTTTGAAATACTCAGACCAGCCTGTCTGGCACCAACAACCATGTCAC
ATTCAAAGTCACAAAATCACCATTCTTTCCCATTCTGATTCTCAGCTTGAACTCCAG
CAAATCGTCTTCACCACATCTAAATGCATTGAGTTGCTGCCAAGTGCTTTGCTGATT
AGCAATTTGTGTTTCCCAGCAATTGAACAGGTGTACCTAAAAAAGTGGTAGCCAG
TGAGTGTGTGTGTATGTATATATATATATATATATATATATATATATATAATGTGTAGGGT
AGCAATTGGAAGTTCACTGTGCAGAAAGGAGATGGAAAGCCTAACAGCCCCTTCC
TTATAAGGACACTTTTCTGGCACTATGGCCCCTAAAATATCCCTGCCATGTCAGGCC
GCGTACACACGACCGATTTTCTCGGCAGAATTCAGCAAGAAGCTCGATGGGAGAC
GTATTCTGCTGAGGAAACCGGTCGTGTGTACACTTTTCGCCGAGCAACCCATCGAG
GAACTCGTCGAGCCAAAAAGAAAGCAGGTTCTCTATTTCCTCGATGGGCAATGGG
AAAATTTGTCTCAACAAGCTTTTTGAATGCCTAACTAGGAACTCAACGGGCAGAAT
GATGAGTTTGTATGTACGCCGCCTCACACTTTTCAGTAGCTTTGCTAATACCGGCAG
AAGATTCAGGTAGTTAATAGCACCACTTTGTAAAATGTAAAAATTAACGTGCCAAA
TTTAAAAATAGTTACCTCTAGTTGGTAAATAGTGAAACTGGAGGTAATAATACCAA
GTATATGCTGGTTATCAGCATGAATGATTAATGCATGGCATGCATTAGACACTCTTTA
TGAATTTCCCGGTGTTTTCACTGAAGATTCTTTTCACGTTACATATTTGTCTGGAAA
ACAAAGCATTTAAAAACAGGTAGTGTGAAGGATGTTTCCAAAGGATAAATGCAGA
TAATTTTGCTTTGTGTGACATGCTCATTATCATTATTATTACTTTATTGTGTTGTGCAT
TTGAAATGTTCCAATAAAATTAAACCCTATTCACATGGCTGTAAAAAAAAGTTTAA
AACCCTTTTTTATTTTTAAATCTGAATGCAGCTGCATAATTTTTAATGGAGACCTTCA
CACATTTAAAAACATGCAGTGGTTTCAGATCTGTAAAATACGGTATATAATCCAAAA
CTTTGTGCCTGAGAACATGCATATATAATTACCTATGCAAATGAAAAAAAAAAAAA
CAGAAAAGTTTTTGTTTTTTTGCATTAAAAAATTACATGGTCATTCTTTGTCCTAAT
TATCAGTAAAAATGTTTACAGCCATGTTACGTCCATGTGAATGTAATCTTAAAGCCA
TTGTTAACTGTTTTGATATGTTTTATTTTCAGCAGAAAGCAAAATGCATGTTCAGGA
TATGCCATCTATCCCCAGCAGGGGGCACATGGAGAGCACATCTGACTTTATGGTTG
ACCCAGCTTATTATGGCAACTTTTACCAACCTTCCCTGTATCCATATTACAACAACC
TCTACAATTATT
CTTCCTACCAAATGGCCATGAGTGGTGAACCATCTGGTGCCAGTGACATAGGTGGA
TCCTCCGTAAAGAATACTATGAGAAACTTCTCGGCTGCTTATGTTCCAGGACAAGC
TGGAAACCAATGGCAGGTAAAGAAATGATAATGTATTGAAGTGTATCTGTCAGTCA
TTTTAACCATTTAAAGTATACACAAAGAATGAGCTGGACCTTGGTCTTTCAGAGCC
TTCTGTGTTGATCTTAATACTCTGAAATACATAATACTGTTCTTAATACTATTAAACA
AAATAAAAATACTCCAGTACTCCACGGTAGTGGGTGTCGATAAAGAGGTGCTTACT
AACCATTGTCTCTCCCTCAATATATAGTAGTTATCGCTAAGGATAAGGTCCTGGTATA

CATTTTAGGGTTCTCCTGTGGAGCCTCACAAACACATATCTCCCCCCAGATCCCCC
CATGAAGGTGACAGGGTCCTGTGAGACTTTTATTCCCTTGAATACACTGTCGACGG
TCCTCAAGTCCTTTGTCTCCCAGGTATTAGTGGCGCTGGTGGAGGGTTCAATTGGT
CGGACTGCTTTCACAGTCAAGCGCTGCGGTTTACCTGCACCACGGGTGCAGCACT
TTGCAACAGATATCTAATATATCCTGTAGGTGTGGCGCACTTTCAGAAAGCTCACCA
AACTCGCAGGTAATAACANNNNNNNNNNNNNNNNNNNNNNNNNNNNNNNN
NNNNNNNNNNNNNNNNNNNNNNNNNNNNNNNNNNNNNNNNNNNNNNNNNN
NNNNNNNNNNNNNGTTTTGCAAGATCGCCACATGGACTTCCGTTCATTCTCTTTAA
AAGTTTTACTGTGTGGAGGCTCTAATTATGCAAGCTTTGGCAACAAGATGCTTCAA
ACCCTCATATGTTGACCCTTACTTTTTTGAGCCTGTTTGCTATGCTGTTGCCCACCC
CTCAGTTTTTTGCTTGGAGACGACTAGCTGTCTGTGAATTTGCTTTACAATTAAGAT
AATTTTGACTTTCCAGTAAATTCCATATCTTAGAGTATGATGCACGGCACTTGTCTC
TCCTATTCTCCTGTCCTCTGTTTCTCCTTATTGCTTGCTACGAAGCTGACATCCCATG
AAGTGTATGGGGTTACATGAGGGTGCACCCAGCAAAGCTTTTACCAATTTCCAATT
ATGTGAAGGTAGCAGCAAATAATCCAAGGTCTGTGCCTTGTACTGCACTCCAAAAT
ATGGAATTTACTGGTGAGTCAAAATTCCCCATTTTTACCATGGTAAATATATGTGATT
TGATGCTTAAAGTTTCTTTGGTAATGCTCTGACGCTGCTTTAGATTCTACACCCCAT
TTATTTTTTAAAACCAGGCAAAGGTGACAATTTGTTCGTTTGGACATGCATGTTGGT
ACCTAACTGCTGATAGATATAATATTGCTTTAAAAAGCCAGTTGATAAATTGTAATA
AGTGTTTTACATCTCTGCAGATGAAAAGTGCTGACAACCGGTTGGCAGGACACAG
TACAAGCTCACAGTACAGAATGCATTCTTACTATCCTCCATACCTGGGGCAGGGTG
TGCCCACTCCTGCCTGTGTTCCACCATTCTTCACATTTGAGGAAAACACAAATTTC
ACAGAGGTCAAGTCAAGTGGTAAGCTTTTGATAATTATATAAAACATTTTGAATGAT
ATAAGGTATCTCAGTTTACAGCTAAATTGACCTTTGATTCTAGGGAAAAAAGCCTC
TGGACAAGAAGTACATTTATTTGGAGGCATTATAAAAAGTGTCAATGTAAAAGGTC
AATTCAATTGTTTTAATTAATTCTACTGTAGCCAGATGTGGTCCTAATCAGGATTACA
GCCTCTTGCCACATTATTTACTGTACAAACTTCTGCCTTTGCTCCAAGCCTGCAGTA
AAGAGGGTGGTGCTAGAACCAGTGAAGACCCCGGGCTGCATTTTGATAGTATTCTT
ATCTTTAAAATAACTATCATCTATAGAGATTTGACTAGCAGGACATACATGTAAAGA
AGGTGAATACAATACATCCTGCAGAGACGTTATTGCCCTGCTTAGCTTAATATCGTC
CACAAATACAGGGATTGAACTGTTTCCCCCATCCTTCAGGNNNNNNNNNNNNNN
NNNNNNNNNNNNNNNNNNNNNNNNNNNNNNNNNNNNNNNNNNNNNNNNNN
NNNNNNNNNNNNNNNNNNNNNNNNNNNNNNNATATTGCTGTTGCTTTCTATT
GCATGCTAGTCAAGTCTAGTCAAAGAGCATAAAGTTTGGCTACCAGAATGTATATTT
TCAATCTATGTTTAGAAGATAGTTTTTTTCATTATTAGGTGAGAAAAGTAATTAATTG
TATTTTGTCTTTATCTTCTACAGTGTTTTCACCACCCAGCAGCCAGGATTCTGGATT
GGTTTCTTTGTCCAGCAGCTCTCCAATCAGCAATGAAAGCACCAAAGGTGTAGCA
GAGTGTGAAGCTCCCACTGATGCCTCCACCTACACAGTAACAAGCATGGCTGATG
ACACC-------AAGATCTGATGTAACCACATGCTGAAACCATCTTTGCAGTAACGGTCT
AGCCATACAATTTAGGTCTACTATAGGGTTATATGTAAAGACTAAGCAGGGCAGTTC
CCCCCCCTTATGATTCATCCCCTTACACATTTTAAGACAATTTTATCATCTGCATGGC
ATATGCCACTTTATCTTAGCATCGCAGGTTTTAAGATTGGCAAAACAGGAACCCTTT
TAGTTTCGGTTAAGTGTCCAGTAACTCAGCACTAACATTTTCATAATGATTTGACAG
TTTGAGCAGTACCCCAACAACTGAACATTACTCAATCTCCCTGCCCTGATTATGCTG

TGTTATCTTCTTTCATTGATAAAAAAAGAAATGAATACTTAGCAAATAAGAATTTCC
ACTTGTGGTTTCATGCATTTTAAAGCCCTAATTGATTGTTTAATAAGGAAGCAATTT
ACCCAAGTGCGTGTTCATTTCACATACTCAGCTTCCCTAGAAATCCTAAATGGTTTA
AAGTAAAACTCCAAGCAACCTACTAATAAACAGTTGAAATTCATATTTAATAATTGT
TTTACCTGCCAAAGGATTTGAAATTCTGGTGAACCATTCATGTGACTNNNNNNNNN
NTGTCCGATGTATTTTTTCATCGGATATCTGATGAAGCTGACTTTTATCAGTCTTGCC
TACACACCATCGGTTAAGAATCCCATCGTGTCCAACGCGGTGATGTAAAACACTAC
GACGTGCTAAGAAAAATTAAGTCCAATGCTTCCGAGCATGCGTCTACTTGATTCTG
AGCATGCGTTGATTTTTGACCAATGGACTTCGCACAGACGATCGTTTTTTCTATTGT
TTTTTTTAACAATAGGAACAATCTAAAACATGTTCTATTTTTTTTCACCGATGGAAA
AAAAAACGATGGGGCCCACACACGATCGGTTCGTCCGATGAAAATGGTCCATCAG
TCTGTTTTCATCAGACAAACCGATCATGTGTACAGGGCTTCACTGTATTAAAAAATA
AATAATCCCGGGTCATATTGCCCACCTTTAGAGCATAATCAGGAGGAGGATGGCCC
ATGACATCACACAACTGGACCAAAGATCCACAGTAAGAAAGTTTTTTATAAGTGG
GGAGAGATTGTAGGGGGGTGGAGGGGGCAAGGCTTACACTTCAGCTTGTGAAATG
ATCAGAAAAATGGACATGATTACAATATTTAAGGAAATGGACAATATTAAAGCCGT
GGGTAACCCTGTTGCCACCATCCTGACTGACAGGCTTTGATCTGAAAATCGAAGG
ACCTAGCCAAACTATTTTCAGACAAACTGGGGCTGGATCCAAGTAGATGCAATCAC
TGTTCAGACATACTAATGCCGCATACACACGATCGGAAATTCTGACAAGAAAAGTC
CGATGTGAGCTTTTGGTCGGAAATTCCGACCGTCTGTAGGCTCCAACAGAATTTTT
CTTTACGAATTTCCGCCAAGAAAAATTGAGAGCTGGTTCTCAAATTTTCCGACGG
AGAATGCAATTTCCGACGGAGAAAAATCCTACGCATGCTTGGAAGCAA

supplementary sequence S7
R403
AAACTAACAATTATTCTTCCTTTTTTTTTTTT.ATCATAATGTTACTGGATAAAAAACA
CAATATTAATTGCTAATTAAGTCCATTAGCAGTTGCAGAATATTAACATATATGCATA
GTTCCAAATGGATTTTAATAGACCTTGTTTTAGTCTAGTTCCACGTGGCTGATTTTT
GCGGTATGACCAAATTAGCAACCCAGGTGTAAAGAAGTCCTGCTGGGATTGGTTTA
TTTCTGCTGAGTTGTAAAAGAAGTAGCTGACAACAGGACTGTGTAAGACCAATCT
CGATCACTTGGGCTTCTCTTTCTCAATTCGCCCGGCTGGCACACCACAAACACCTT
CATTAATGTCCTTTTATAATGTGATTTATTTATGGAAGTGACAAAGCTTCACATTGCA
CCAC

supplementary sequence S8
R1092
TTGTGGAGGATTGTTACCCAATTGTATTATTGTGGGAAGGACATTAGACACGATGTC
CCCTGTTCCTCCTACTAACTCCCACCCAGGTCTTCTCTCAACATTCTTCACGCTACA
ATTTGTCTCAGAGCAAGAATCACTTTAGCTTGATGCGTTGTTTGCTGACTAATTGTA
ATTTTTATTTTTTTACAGTGATCTAAAAAAAAAATAAAAAAATGCCTAACAGCGAGG
AGCCATTAAGCAAGCCTCGTAAACCTGGGGTACAGTCCTCGGGGGCTCAATGGAA
GAAATCCCCACGCATGCCAAAATGTTCTCGTTGCAGAAACCACGGCTATTCGTCGC

TACTAAAGGGGCACAAGCGTTTCTGTATGTGGCGGGAATGTCAGTGTAAAAAATG
CAGTCTTATTGCGGAGAGACAGCGGGTAATGGCGGCCCAGGTAGGAAAAACAGTA
TAACTCTATATACACCTTAATGTTCATTTGCCTATGCATTATAATTTCTTATAGTGTGC
GTGTGTGTGTGCGTGTGTGTGTGTGTGCGTGTGTGCTAGCAGAATAAAGAGACCC
AGTCATTTTTCTGTAAACTGTCACTAGAGATACTAATTTCTTCCATACTCCTGTAAAT
GCTAGATCTTTTCTCTATCAAGGTGCAATATGCTCCAATTAATGGCGTGCTCAGCGC
TGTAAGTGAAATCCAACATAAGAACACATATAAGAAAAAAATGTGCACACTATACT
ATGTGAACAAATGCAAGCATACCTGTGAATGTAAGGAAAAGACTTCACATATAATG
GAACCATTCACCTCATGTCTCCACCCTTGATTGAAAAATATAATTTGACCGCCGTGC
CCACTTGTAG.TCAGTAGCACTTGTGTTTTTATAGGTTGGTATGCTGGTAATCACTAT
TTAACATCTCTGTCCAATACAACAATAATTAGTCTATTGCCACAGGTGTCTAGGTTT
CATATGATTAGCTGTAGTCATTTTGTGTATTCGTTTTGGGTGGAGAGCGATATTATTT
TTGTTTTGGGTGGAGAGCAATATTATTTTTGTTTTGGGTGGAGAGCCATATTATTTTT
GTTTTGGGTGGAGAG

supplementary sequence S9
R940
GGGCAGAATGATGAGTTTGTATGTACGCCGCCTCACACTTTTCAGTAGCTTTGCTAA
TACCGGCAGAAGATTCAGGTAGTTAATAGCACCACTTTGTAAAATGTAAAAATTAA
TGTGCCAAATTTAAAAATAGTTACCTCTAGTTGGTAAATAGTGAAACTGGAGGTAA
TTAATACGTATATATATGCTGGTTATCAGCATGAATGAATCGCGCATGGCATGCATTG
GGCACTCTTTATGAATTTCCCAGTGTTTTCACTGAAGATTCTTTTCACGTTACATATT
TGTCTGGAAAACAAAGCATTTAAAAACAGGTAGTGTGAAGGATGTTTCCAAAGGA
TAAATGCAGATAGTTTTGCTTTGTGTGACGTGCTCATTATCATTATTATTACTTTATTG
TGTTGTGCATTTGAAATGTTCCAATAAAATTAAACCCTATTCACATGGCTGTAAAAA
AAAGTTTAAAACCCTTTTTTATTTTCAAATCTGAATGCAGCTGCATAATTTTCAATT
GAGACCTTCACACATTCAAAAACATGCAGTGGTTTCAGATCTGTAAAATATGGTATA
TAATCCAAAACTTTGTGTCTGAGAACATGCATATATAATTACCTATGCAAAT.......GGA
AAAAAAAAACAGGATAGTTTTTT.TTTTTTTGCATTCAAAAATTATCTGGTCATATCT
TGTCCTAATTATCAGTAAAAATGTTTACAGCCATGTTACGTCCATGTGAATGTAACC
TTAACCTTAAAGCTATTGTTAACTGTTTTGATATGTTTTATTTTCTGCAGAAAGCAG
AATGCATGTTCAGGATATGCCATCTATCCCCAGCAGGGGGCACATGGAGAGCACAT
CTGACTTTATGGTTGACCCAGCTTATTATGGCAACTTTTACCAACCTTCCCTGTATC
CATATTACAACAACCTCTACAATTATTCTTCCTACC
