# Supplementary figures and images for: Genome Survey of Male Rana dybowskii to Further Understand the Sex Determination Mechanism
Source: Animals (Basel). 2024 Oct 14;14(20):2968. doi: 10.3390/ani14202968 (PMC11503867; doi:10.3390/ani14202968)

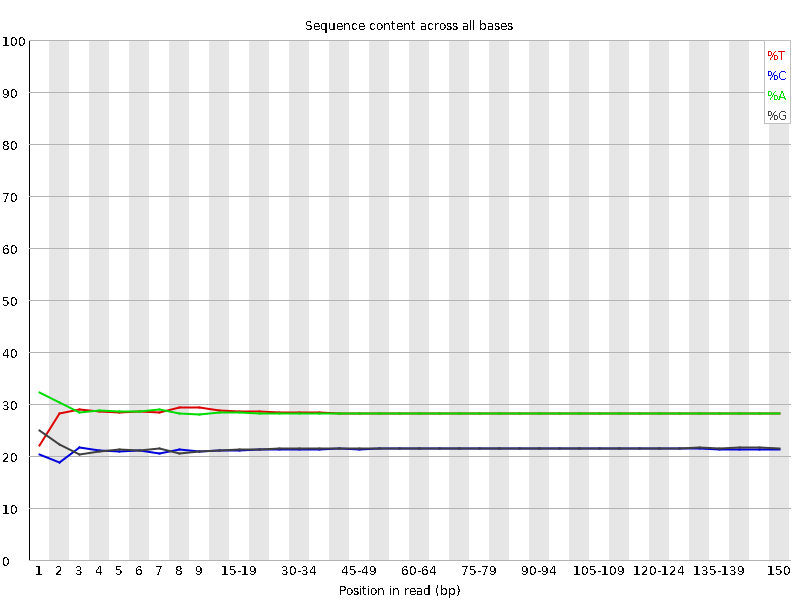

Supplement: Supplementary file 1 [file animals-14-02968-s001.zip › Supplementary Figure S1 a.png]

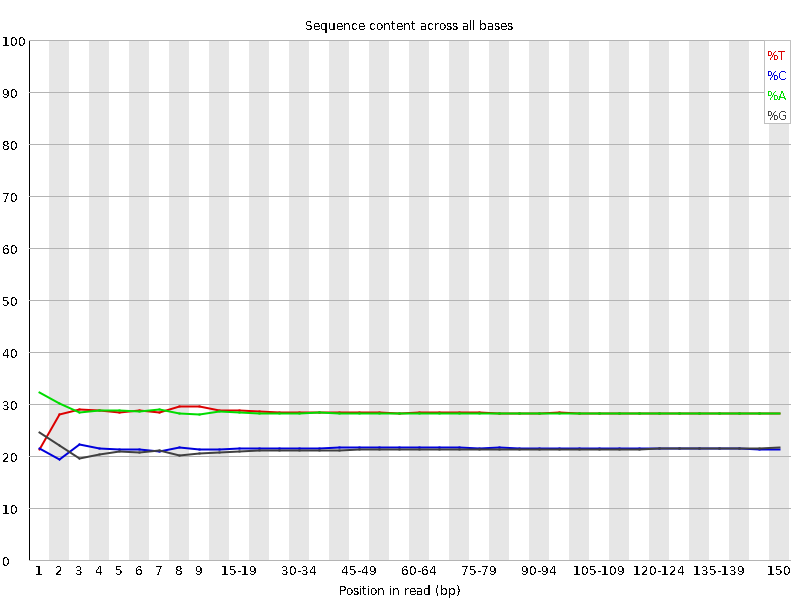

Supplement: Supplementary file 1 [file animals-14-02968-s001.zip › Supplementary Figure S1 b.png]

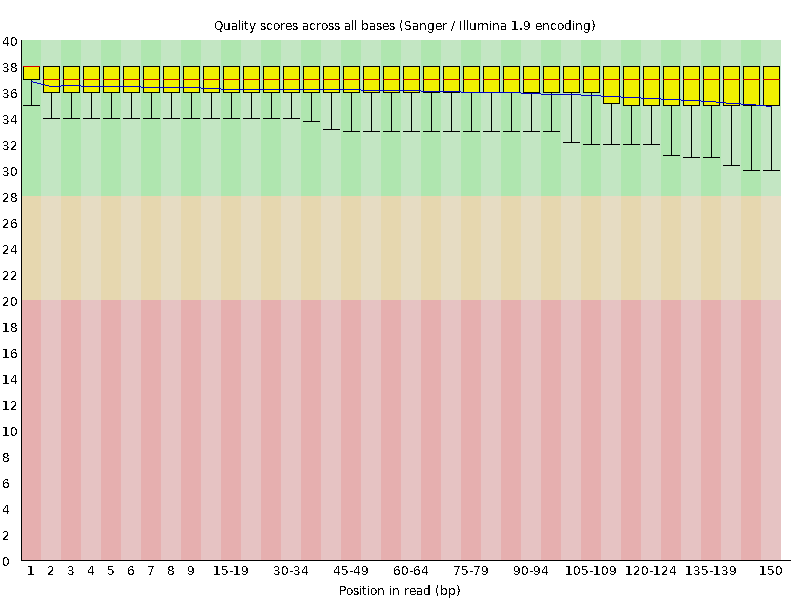

Supplement: Supplementary file 1 [file animals-14-02968-s001.zip › Supplementary Figure S2 a.png]

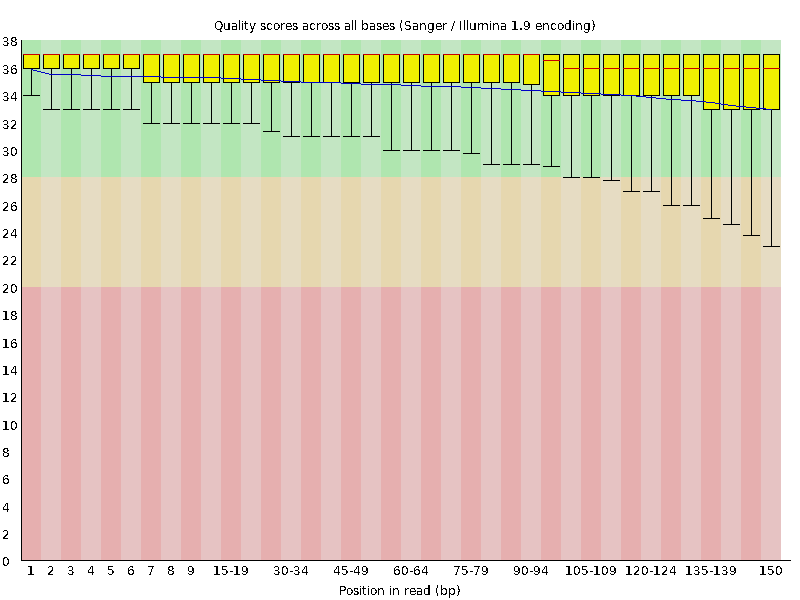

Supplement: Supplementary file 1 [file animals-14-02968-s001.zip › Supplementary Figure S2 b.png]

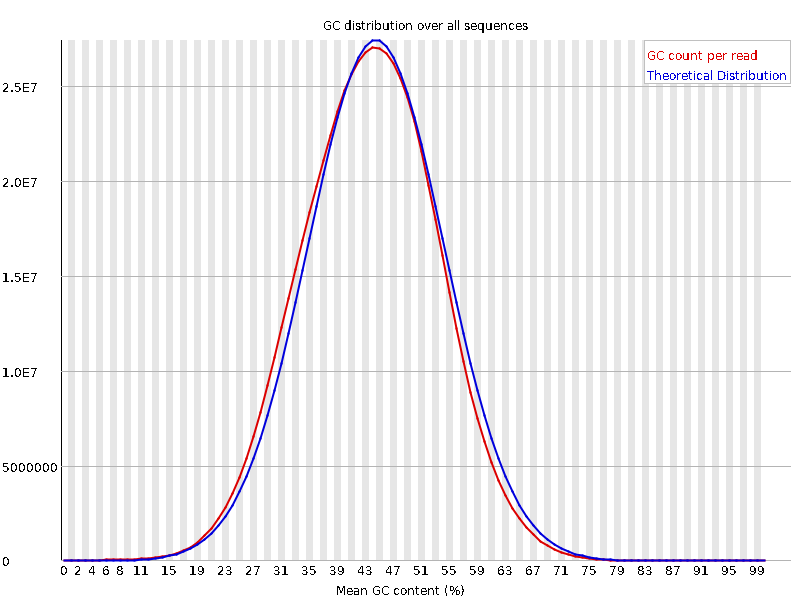

Supplement: Supplementary file 1 [file animals-14-02968-s001.zip › Supplementary Figure S3 a.png]

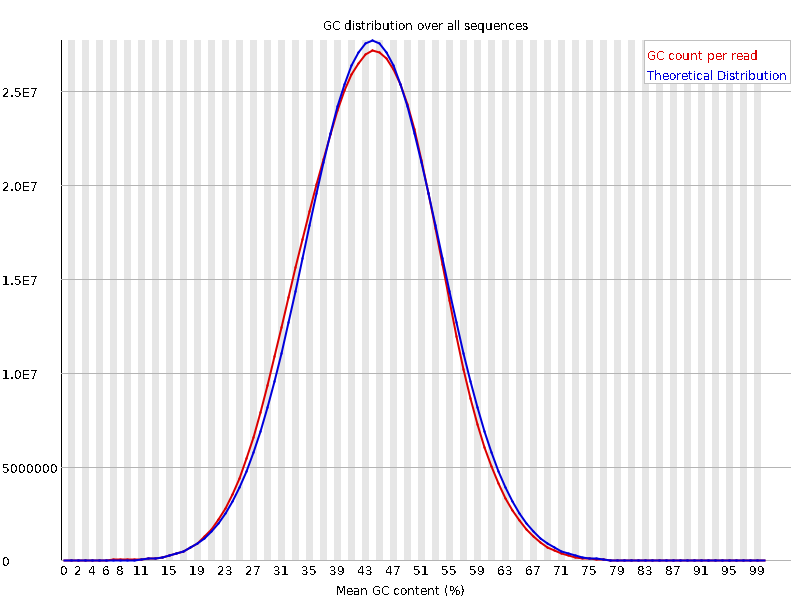

Supplement: Supplementary file 1 [file animals-14-02968-s001.zip › Supplementary Figure S3 b.png]
